# HDL and CER-001 Inverse-Dose Dependent Inhibition of Atherosclerotic Plaque Formation in apoE^-/-^ Mice: Evidence of ABCA1 Down-Regulation

**DOI:** 10.1371/journal.pone.0137584

**Published:** 2015-09-03

**Authors:** Claudine Tardy, Marine Goffinet, Nadia Boubekeur, Guy Cholez, Rose Ackermann, Gavin Sy, Constance Keyserling, Narendra Lalwani, John F. Paolini, Jean-Louis Dasseux, Ronald Barbaras, Rudi Baron

**Affiliations:** 1 Cerenis Therapeutics SA, Labege, France; 2 Cerenis Therapeutics Inc., Ann Arbor, Michigan, United States of America; University of Padova, ITALY

## Abstract

**Objective:**

CER-001 is a novel engineered HDL-mimetic comprised of recombinant human apoA-I and charged phospholipids that was designed to mimic the beneficial properties of nascent pre-ß HDL. In this study, we have evaluated the dose-dependent regulation of ABCA1 expression *in vitro* and *in vivo* in the presence of CER-001 and native HDL (HDL3).

**Methods and Results:**

CER-001 induced cholesterol efflux from J774 macrophages in a dose-dependent manner similar to natural HDL. A strong down-regulation of the ATP-binding cassette A1 (ABCA1) transporter mRNA (- 50%) as well as the ABCA1 membrane protein expression (- 50%) was observed at higher doses of CER-001 and HDL_3_ compared to non-lipidated apoA-I. *In vivo*, in an apoE^-/-^ mouse “flow cessation model,” in which the left carotid artery was ligatured to induce local inflammation, the inhibition of atherosclerotic plaque burden progression in response to a dose-range of every-other-day CER-001 or HDL in the presence of a high-fat diet for two weeks was assessed. We observed a U-shaped dose-response curve: inhibition of the plaque total cholesterol content increased with increasing doses of CER-001 or HDL3 up to a maximum inhibition (- 51%) at 5 mg/kg; however, as the dose was increased above this threshold, a progressively less pronounced inhibition of progression was observed, reaching a complete absence of inhibition of progression at doses of 20 mg/kg and over. ABCA1 protein expression in the same atherosclerotic plaque was decreased by-45% and-68% at 50 mg/kg for CER-001 and HDL respectively. Conversely, a-12% and 0% decrease in ABCA1 protein expression was observed at the 5 mg/kg dose for CER-001 and HDL respectively.

**Conclusions:**

These data demonstrate that high doses of HDL and CER-001 are less effective at slowing progression of atherosclerotic plaque in apoE^-/-^ mice compared to lower doses, following a U-shaped dose-response curve. A potential mechanism for this phenomenon is supported by the observation that high doses of HDL and CER-001 induce a rapid and strong down-regulation of ABCA1 both *in vitro* and *in vivo*. In conclusion, maximally efficient HDL- or CER-001-mediated cholesterol removal from atherosclerotic plaque is achieved by maximizing macrophage-mediated efflux from the plaque while minimizing dose-dependent down-regulation of ABCA1 expression. These observations may help define the optimal dose of HDL mimetics for testing in clinical trials of atherosclerotic burden regression.

## Introduction

The protective effect of high density lipoprotein (HDL) against atherosclerosis is in large part attributed to its ability to mobilize cholesterol from the vessel wall, in particular from lipid-rich atherosclerotic plaques, and to transport it to the liver where it is excreted from the body in the form of bile acids or unesterified cholesterol [1, 2].

The population of HDL particles consists of particles of various sizes, depending on how much cholesterol each particle has mobilized and is carrying for transport to the liver for elimination. The newly formed smallest particles (also called pre-β HDL) are essentially “empty” and have the greatest ability to mobilize cholesterol [3, 4]. A natural pre-β HDL is a lipoprotein consisting of apolipoprotein A-I (apoA-I) and phospholipids interacting together to form a small discoidal particle. In a normal individual, in terms of cholesterol content, pre-β HDL particles represent only 10% of the total HDL-cholesterol [5–9]. These small particles increase in size as they accumulate cholesterol, creating larger, “full,” and mature alpha-HDL particles capable of delivering that collected cholesterol to the liver for elimination.

Reverse lipid transport or more specifically Reverse Cholesterol Transport (RCT) describes the transfer of cholesterol from non-hepatic cells to the liver. The first step is the removal of cholesterol from arteries by the nascent HDL particle in a process termed “cholesterol efflux.” Cholesterol is a membrane constituent that maintains structural domains that are important in the regulation of vesicular trafficking and signal transduction. In most cells, cholesterol is not catabolized. Thus, the regulation of cellular sterol efflux plays a crucial role in cellular sterol homeostasis. Cellular sterol can efflux to extracellular sterol acceptors by both non-regulated, passive diffusion mechanisms as well as by an active, regulated, energy-dependent process mediated by the ATP-binding cassette A1 (ABCA1) transporter [10–13].

In the second step, cholesterol is esterified to form a cholesteryl ester that is more tightly associated with the HDL particle as it is carried in the blood; this process is called “cholesterol conversion/esterification” [14]. The third step is the transport and delivery of that esterified cholesterol to the liver in a process termed “cholesterol transport” [15, 16].

The final step is the recognition, transformation and disposal of cholesterol by the liver in a process termed “cholesterol elimination” [17]. It is of note that recent studies in mice have revealed that the intestine could also act as an excretory organ for the cholesterol. In humans, this mechanism was recently described using intestinal explant [18]. However, the exact contribution of this trans-intestinal cholesterol excretion pathway (TICE) in the overall cholesterol homeostasis still needs to be established [19, 20]. RCT is therefore in humans the main natural mechanism capable of transporting cholesterol from vessel wall plaque back to the liver to be eliminated and consequently to reverse accumulation of cholesterol in plaque.

Consequently, one can understand that a low number of HDL particles could result in accumulation of lipids and cholesterol throughout the body and, in particular, in the vessel wall in the form of atherosclerotic plaque. This phenomenon has been identified in patients with ultra-low circulating HDL particle numbers due to genetic defects (Familial Primary Hypoalphalipoproteinemia) such as apoA-I, ABCA1, and LCAT deficiencies. Such a disease could be amenable to treatment with replacement therapy consisting of an engineered human apoA-I-containing pre-ß HDL particle. Indeed, restoring the flux of cholesterol through the RCT pathway, by providing apoA-I and the missing cholesterol elimination capacity in the form of very efficient and functional pre-β HDL particles, has been demonstrated to lead to removal of accumulated cholesterol from the body into the feces and to reduce carotid artery wall thickness in FPHA patients[21].

Previous experiments have shown that the infusion of HDL purified from plasma into cholesterol-fed rabbits limited the extent of aortic fatty streaks and lowered lipid deposition in the arterial wall [22–24].

Short-term infusion of HDL-mimetics in animal models as well as in humans has shown promising effects on the plaque size and morphology [25, 26]. The intravenous administration of recombinant apolipoprotein A-I_milano_/phospholipid complex (ETC-216), the first randomized trial of HDL infusion in humans, produced significant reduction of coronary atherosclerosis as measured by IVUS [27]. Recently, in a dose-response trial of a new HDL mimetic (CER-001), although the primary clinical endpoint was not reached, the analysis of the modified Per Protocol population has shown improvement of the coronary plaque volume as analyzed by IVUS technique at one dose level which was nominally statistically significant not only versus baseline but also versus placebo [28]. Interestingly the dose which demonstrated the greatest efficacy was the lowest dose tested in the trial, 3 mg/kg, and the magnitude of the IVUS effect as well as of cholesterol mobilization observed with the CER-001 3 mg/kg dose was similar to that seen with ETC-216 15 mg/kg in the apolipoprotein A-I_milano_ trial, confirming the 5–7-fold greater potency of CER-001 versus ETC-216 seen in preclinical experiments. Also of note, the trend to lesser efficacy with a higher dose was also noted in the apoA-I_milano_ trial.

The ABCA1 transporter mediates the cellular efflux of lipids, in particular phospholipids and cholesterol, from peripheral tissues to lipid-poor apoA-I in the plasma, resulting in the formation of pre-β HDL particles. In preclinical models the role of both hepatic and extrahepatic ABCA1 in the assembly of the HDL particle has been demonstrated. In humans, homozygous mutations in the ABCA1 gene leading to defective or non-functional ABCA1 receptors result in Tangier disease, characterized by profoundly decreased HDL-C, apoA-I and apoA-II levels, reduced total and low-density lipoprotein cholesterol (LDL-C) and apoB, and elevated plasma triglyceride levels. With ABCA1 deficiency, apoA-I is rapidly cleared before it is able to acquire cholesterol. The cholesterol storage disorder that occurs with ABCA1 mutations might thus possibly be more a consequence of HDL deficiency than a direct consequence of dysfunctional ABCA1 [29].

ABCA1 was previously described *in vitro* to be down-regulated by infusion of HDL[30]. All together, we hypothesized in the present publication that natural HDL as well as the HDL-mimetic CER-001 could trigger an ABCA1 down-regulation and thus explain the phenomenon that increasing the dose *in vivo* beyond a critical threshold could attenuate its efficacy in removing cholesterol from the vessel wall.

## Material and Methods

### Ethics Statement

The animal studies were conducted according to the recommendations of European Directive 2010/63/UE, and protocol approvals were obtained from institutional ethic committees (Midi Pyrénées ethic committee, France).

### Preparation of CER-001

Recombinant human pre-pro-apolipoproteinA-I (NP_000030.1, 267 residues) was expressed in CHO cells using the GPEx system (for “gene product expression”). This system utilizes replication-defective retroviral vectors, derived from Moloney murine leukemia virus (MLV) and pseudo- typed with vesicular stomatitis virus G protein (VSV-G), to stably insert single copies of genes into dividing cells (Catalent Pharma Solution, Madison WI) [31]. The resulting transgene construct pCS-apoA-I-WPRE was used on to CHO cells. A stable cell line was created for the production of recombinant apoA-I (Catalent Pharma Solution, Madison WI). The recombinant protein was produced and custom purified by Novasep (France). The purified protein was formulated into CER-001 (HDL mimetic particle) preparations by complexation with phospholipid containing egg sphingomyelin and dipalmitolyphosphatidyl glycerol (97:3). The protein-to-phospholipid ratio was 1:2.7 in the solution. The doses of CER-001 were defined by the quantity of human apoA-I present in the infusion.

### Purification of human LDL, HDL_2_ and HDL_3_

Classes of lipoproteins were isolated from plasma of normolipidemic healthy human donors provided by blood donation center (Etablissement Français du Sang; http://www.dondusang.net/rewrite/site/39/french-blood-service.htm?idRubrique=1092). The lipoproteins were obtained by sequential flotation ultracentrifugation in KBr solution (VLDL, d = 1.006 g/mL; LDL, 1.006 < d < 1.063 g/mL). HDL_2_ were first isolated (110,000 x g for 40 h) at d = 1.125 g/mL followed by HDL_3_ (110,000 x g for 40 h) at d = 1.19 g/mL. Before use, the lipoproteins were extensively dialyzed against phosphate-buffered saline.

### LDL oxidation

PBS-dialysed LDL (2mg/mL) were oxidized using CuSO4 (5μM final concentration) (C8027, Sigma Aldrich) for 4H at 37°C. The reaction was stopped by adding EDTA (100μM final concentration) (#20302.236, Prolabo). The oxidized LDL were dialysed against 2x1L PBS for 0.5H before use.

### Animals

C57Bl/6J mice, 9 weeks old weighing approximately 18–20g were purchased from Janvier (France). Animal housing and care were in compliance with the recommendations of Directive 86/609/EEC, and protocol approvals were obtained from institutional ethic committees.

### ApoE^-/-^ mouse “flow cessation model”

*The* left carotid arteries of apoE^-/-^ mice (9–10 week old) were ligatured, and mice were placed on a High Cholesterol Diet (HCD) for one week prior CER-001 and HDL3 injections. Then these mice were given (retro orbital injection) CER-001 or HDL3 every other day at the indicated doses (from 2 to 50 mg/kg in 0.5% carboxymethyl cellulose (CMC), 0.2% Tween80) for 2 weeks (8 injections) on the same HCD.

### Carotid cholesterol measurement

The samples were first weighted and lipid extracted overnight at 4°C in Chloroform:Methanol (2:1) with addition of stigmasterol as internal standard. Samples were assayed for total cholesterol after saponification in methanolic KOH [32]. Carotid samples were analyzed by HPLC [33].

### Membrane/Cytosol separation

Tissue sample was homogenized by sonication (2 x 10s at 30% of amplitude using the Digital Sonifier BRANSON) in 20 mM Tris-HCl,150 mM, NaCl, 1 mM EDTA, 2 mM MgCl_2_, 1X protease inhibitor and centrifuged at 800 x g for 5 min at 4°C. Supernatant was centrifuged for 1 h at 100,000 x g (4°C) and the pellet (membrane fraction) was solubilized in the same lysis buffer complemented with 1.2% Triton X100. Unsolubilized membrane fractions were removed by Centrifugation for 5 minutes at 14,000 x g.

### ABCA1 determination in ligatured carotids

Ligatured carotids first extracted with Chloroform:Methanol, were solubilized in NAOH 0.1N (100μL/carotid). The solution is briefly sonicated and centrifuged at 15,000 x g for 10 minutes. The protein concentration was determined with Bradford assay and 40μg of sample were loaded on SDS-PAGE. The ABCA1 expression (ab7360 from Abcam—dilution 1/1000) was quantified using imageJ software. Calnexin expression was used as a loading control for each sample (ab10286 from Abcam—dilution 1/1000). The different samples were quantified and normalized to the same reference sample loaded each time on the SDS-PAGE.

### Assay for Cholesterol Efflux

J774 mouse macrophages obtained from ATCC (N° TIB-67) were grown in Dulbecco's modified Eagle's medium (DMEM, Invitrogen) supplemented with 10% FBS (foetal bovine serum, Invitrogen), 100 units/mL penicillin G (Invitrogen) and 100 units/mL streptomycin (Invitrogen) at 37°C with 5% CO_2_. Cells were seeded on 24-well plates at 60 000 cells/well. The day later, oxidized LDL (50μL, 12.5μg) was mixed with [^3^H] cholesterol (1μCi, Perkin Elmer) in DMEM 2.5% FBS for 15 minutes and added to J774 cells in 500μL DMEM 2.5% FBS for 24h. Then medium was removed and J774 macrophages were equilibrated an additional 16h with 0.5mL DMEM medium/well in presence or not of cAMP (300μM). The efflux was induced by adding CER-001, HDL_3_ or human recombinant apoA-I (25μg/mL) for 6h in 250μL DMEM without FBS. After the incubation, the radioactivity in medium (0.25mL) and the intracellular [^3^H] cholesterol (extracted with 0.5 mL hexane-isopropanol (3:2)) were measured by liquid scintillation counting. ABCA1 specific efflux was determined by comparison of efflux in presence of absence of cAMP.

### QPCR analysis

Cells were homogenized in 1ml TRIZOL, and the mRNAs were extracted with the RiboPure kit (Ambion) and reverse transcribed with the High capacity RNA to cDNA kit (Applied Biosystems) according to the manufacturer protocols. The mRNA integrity was verified with the Agilent 2100 Bioanalyzer. Real time quantitative PCR for ABCA1 used Taqman probe Mm00442646.m1 and Taqman probe Mm00446968.m1 for the internal standard HPRT-1. Each experimental condition was performed in triplicate.

#### Statistical analysis

All statistical analyses were performed using T-test protocol with 95% confidence using Prism software (Graphpad).

## Results

### Attenuated inhibition of atherosclerotic plaque progression in the ligatured carotids of apoE^-/-^ mice at high HDL doses is associated with down-regulation of ABCA1

**Fig 1:**
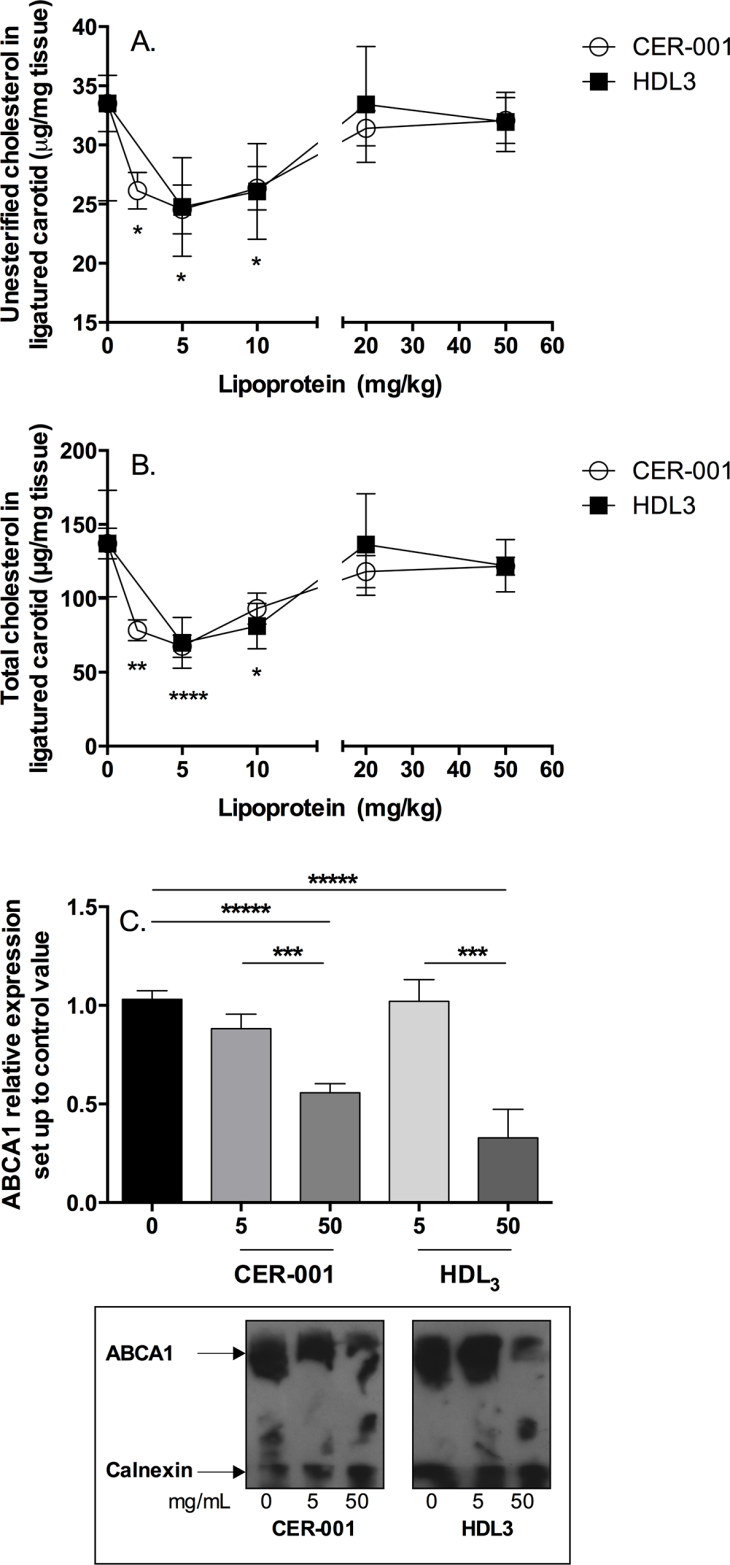
Effect of dose-response of CER-001 and HDL3 in atherosclerotic plaque progression in ligatured carotid of apoE^-/-^ mice. The left carotid of apoE^-/-^ mice (n = 12) was ligatured, fed with HCD and treated (retro-orbital injection) every second day with different concentrations of CER-001 or HDL3 (8 infusions). The carotids were lipid extracted and cholesterol concentrations were determined by HPLC. Panel A; unesterified cholesterol. Panel B; total cholesterol. Panel C; protein level of ABCA1 was measured in the ligatured carotids using Western blot analysis as described in material and methods section. The data represent the means of ABCA1 expression from at least 5 different carotids. Representative Western-blot for carotid analysis was resolved with anti-ABCA1 antibody (1/1000 dilution) and anti-Calnexin antibody (1/1000). * p<0.05, ** p<0.01, ***p<0.005, ****p<0.001, *****p<0.0001

There is evidence in the literature for a trend toward lesser efficacy with higher doses of different HDL mimetics (i.e. ETC-216, CER-001) in clinical trials [34, 35]. We assessed the CER-001 and HDL3 dose response *in vivo* by assessing plaque formation in ligatured carotid of apoE^-/-^ mice fed with a high cholesterol diet (HCD). The left carotid of apoE mice was ligatured [36–38], and groups of mice (n = 12) were subsequently fed with HCD and treated (retro-orbital injection) every second day with different dose levels of CER-001 or HDL3 for two weeks. After 8 infusions, the carotids were excised and lipid-extracted, and vessel cholesterol content was determined by HPLC. We observed a gradually greater decrease for unesterified and total cholesterol content in ligatured carotids as the dose of CER-001 or HDL3 increased from 2 to 5 to 10mg/kg (Fig 1A and 1B). For dose levels >10mg/kg, the cholesterol content was not decreased and instead was at the same level of untreated mice. The ABCA1 protein content in the ligatured carotids paralleled that of the cholesterol content in a similar dose-related manner (Fig 1C). In the same experiment, we observed that at doses for which plaque formation in carotid (5mg/kg of CER-001 and HDL3) is maximally inhibited, carotid artery ABCA1 protein levels are similar to those of untreated animals. In contrast, at the 50mg/kg dose of CER-001 or HDL3, the carotid cholesterol content of plaque burden is similar to that of untreated animals, and the carotid artery ABCA1 protein level is decreased by half compared to untreated animals. Thus, down-regulation of carotid ABCA1 after treatment with elevated concentrations of CER-001 or HDL3 appears to have attenuated the efficacy of the infused HDL to inhibit plaque formation in the apoE^-/-^ mouse ligatured carotid model; however, at lower doses, increasing the dose of CER-001 and HDL3 up to that observed threshold slowed progression of carotid plaque while maintaining normal levels of ABCA1 expression

### CER-001 and HDL3 decrease ABCA1 mRNA and protein levels in J774 macrophages

**Fig 2:**
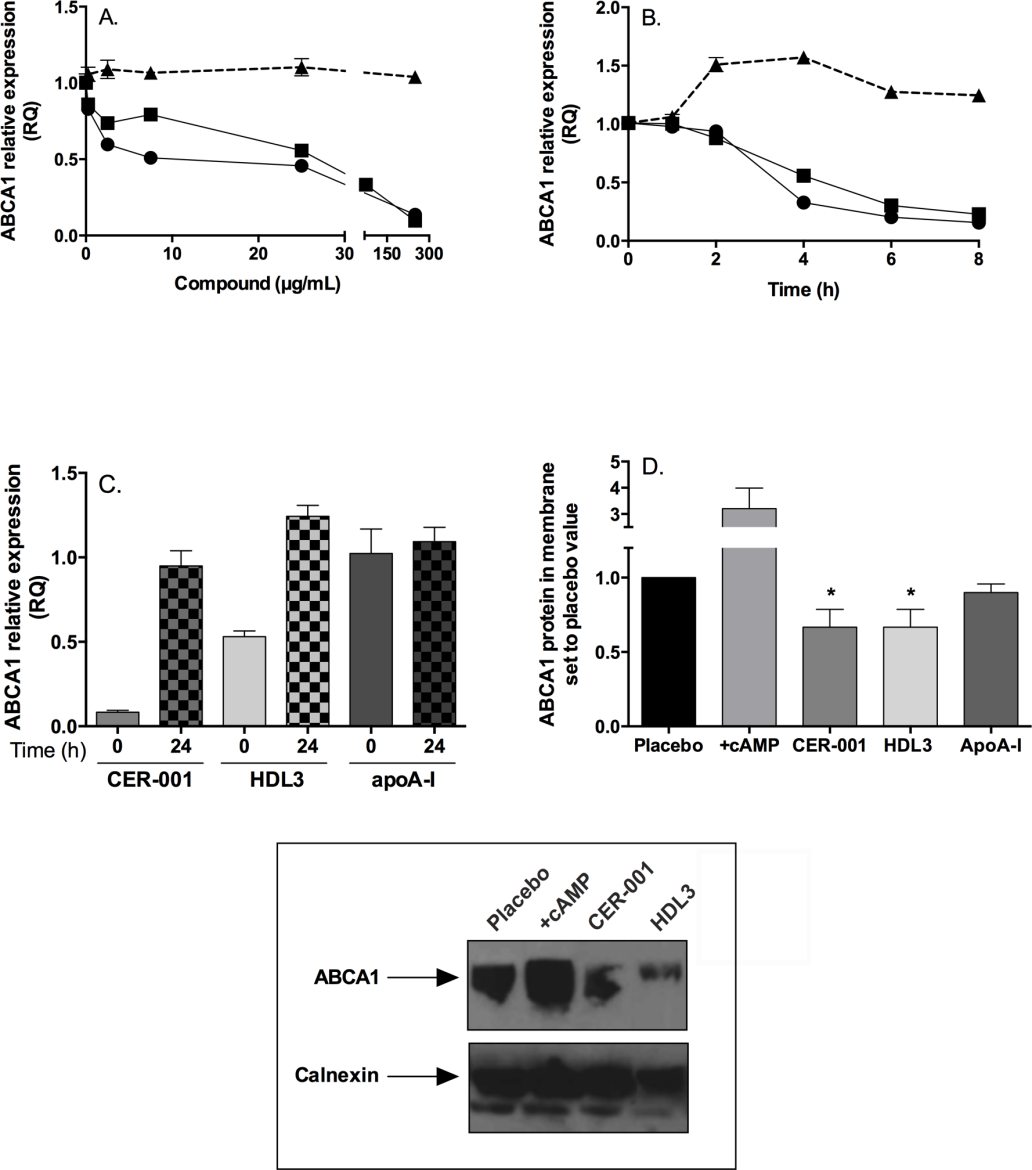
ABCA1 mRNA and protein decrease in J774 macrophages following CER-001 and HDL3 incubation. Panel A; ABCA1 mRNA level in J774 macrophages was determined in presence of different concentrations of CER-001, HDL3 or delipidated apoA-I for 24h. Panel B; Kinetic of ABCA1 mRNA level at a fixed concentration (250μg/mL) of CER-001, HDL3 or delipidated apoA-I. The qPCR data represent the means of triplicate determinations from a single experiment that is representative of three such experiments. Panel C; J774 macrophages were treated for 24 h in presence of CER-001, HDL3 or delipidated apoA-I at 250μg/mL (time 0). Compounds were removed and replaced with fresh medium for 24 h (time 24) and ABCA1 mRNA level was determined by qPCR. Panel D; The relative membrane protein expression of ABCA1 was measured by Western blot analysis for macrophages treated for 6h with CER-001, HDL3 or apoA-I at 250 μg/ml. cAMP was used for 24h at 300μM. Membrane protein loading for each sample was verified by Western blot using rabbit anti-Calnexin (1/1000 dilution). Membrane fractions were obtained after ultracentrifugation as described in Material & Method section. The data represent the means of triplicate determinations from a single experiment. * p>0.05.

ABCA1 has been shown to be cardioprotective in mouse models[39] and human disease[40, 41]. Loss of function mutations of the protein in Tangier disease increases the severity of atherosclerosis[42]. We have determined the incremental effect of different concentrations of CER-001, HDL3 or delipidated apoA-I on ABCA1 mRNA level from J774 macrophages over 6 h (Fig 2A) or the effect of a fixed concentration (250 μg/mL) of CER-001, HDL3 or delipidated apoA-I (Fig 2B) on ABCA1 mRNA level at different time points. We observed that at CER-001 or HDL3 concentrations > 20 μg/mL, ABCA1 mRNA expression decreases to half that of controls. Delipidated apoA-I did not change the ABCA1 mRNA level even at concentrations up to 300μg/mL (Fig 2A). Upon testing a fixed high concentration of CER-001 or HDL3 (250 μg/ml), half of the reduction mRNA amount was observed at 4h, with a plateau at 8h. In a separate experiment, we observed that the mRNA levels returned to pre-treatment control levels after 24 hours of washout (Fig 2C). In presence of delipidated apoA-I, ABCA1 mRNA is not diminished; instead there is a trend to an increase in the ABCA1 mRNA level at 4h.

Because the half-life of ABCA1 is fast (1h)[43], it was expected that the down-regulation of ABCA1 mRNA should be seen at the protein level. ABCA1 protein content was measured in macrophages treated for 6h with CER-001, HDL3 or apoA-I. Membane/cytosol were isolated by ultracentrifugation, resolved by Western blot and probed against ABCA1 (Fig 2D). As already described in the literature[44] the addition of cAMP boosted the ABCA1 protein level[44] compared to untreated macrophages. In the presence of high dose (250 μg/mL) CER-001 or HDL3, the ABCA1 protein level in the membrane was significantly decreased by about 30%. Delipidated apoA-I did not affect macrophage ABCA1 protein content.

### CER-001 and HDL3 decrease ABCA1-specific efflux in J774 macrophages

**Fig 3:**
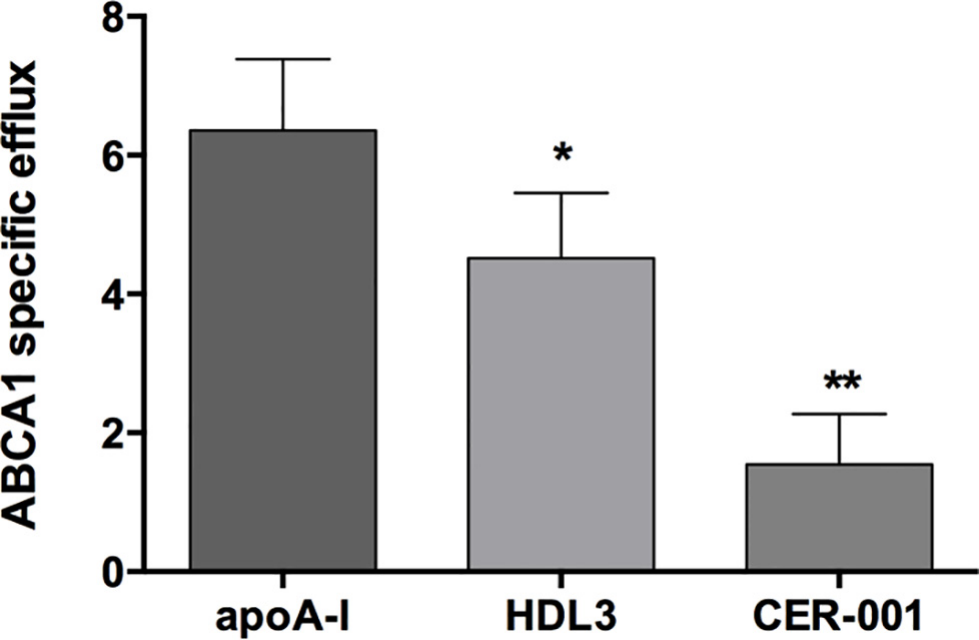
cAMP stimulated-ABCA1 specific efflux decrease in J774 macrophages following CER-001 and HDL3 incubation. J774 macrophages were incubated with cAMP to increase the ABCA1 expression and then CER-001 and HDL3 at 25 μg/ml were added in the cell culture medium and the specific cAMP cholesterol efflux was determined. apoA-I (25 μg/ml) was used as reference for specific ABCA1-cholesterol efflux in the experiment. ** p<0.01, ***p<0.001.

As described above ABCA1 mRNA level is decreased after treatment with CER-001 or HDL3. A similar assessment was observed after stimulation of J774 macrophages with cAMP and increasing concentrations of CER-001 or HDL3 (S1 Fig). The up-regulation of ABCA1 by cAMP has previously been shown to increase the cholesterol efflux in macrophages[45] (named: ABCA1-specific efflux); therefore, we postulated that the down-regulation of ABCA1 should decrease this ABCA1-specific efflux. Following stimulation of macrophages with cAMP, CER-001 and HDL3 or free-delipidated apoA-I as a control were added in the cell culture medium, and the ABCA1-specific cholesterol efflux was determined (Fig 3). We observed a decrease in ABCA1-specific cholesterol efflux with both CER-001 and HDL3 (25 μg/ml) of a similar magnitude, compared to no effect with the free-delipidated apoA-I. Thus, the ABCA1 mRNA down-regulation induced by CER-001 and HDL3 is associated with a reduction in the ABCA1-specific cholesterol efflux from macrophages.

## Discussion

The HDL mimetic CER-001 as well as purified HDL induced down-regulation of ABCA1 in a dose-dependent manner. This observation needs to be put in perspective of the following previous observations. Historically, ABCA1 in macrophages was described *in vitro* to be down-regulated by incubation with HDL[30]. *In vivo*, a scrutiny analysis of the first randomized trial of HDL infusion in humans using an apoA-I_milano_/phospholipid complex has shown at the higher dose, 45 mg/kg, a trend to less efficacy than at the lower dose, 15 mg/kg [27]. The same observation can be applied for the CER-001 clinical trial where a promising effect on plaque regression seems to be detected at only the lowest dose of 3 mg/kg [28]. *In vivo*, in the present manuscript, we also observed that a significant decrease of the carotid plaque burden of the left carotid of high-cholesterol diet apoE^-/-^ mice took place only at the lower doses of CER-001 and HDL3. Conversely, the ABCA1 protein expression in carotid atherosclerotic plaque is decreased only at the highest doses, whereas no down-regulation was observed at low doses.

ABCA1 mRNA and protein level are decreased after treatment with CER-001 or HDL3. Further analysis needs to be done, but one can hypothesize that the down-regulation of ABCA1 could take place when a low level of cholesterol is reached in the macrophages. Interestingly, *in vitro* the well-known treatment of the macrophages using cAMP, which induced a strong increase in the ABCA1 expression, did not impede the HDL-mediated down-regulation of ABCA1.

Today, another ABC transporter, ABCG1 seems to be also involved in the cellular cholesterol efflux process mediated by HDL particles. ABCG1 seems to act in addition of ABCA1 for cholesterol efflux with an appetency for larger HDL particles than for ABCA1 [46]. Unfortunately, in humans, the real effect of ABCG1 on global cholesterol efflux has not been described as well as its effect on the development of the atherosclerotic plaque burden. But further study of a potential down-regulation of ABCG1 by the HDL identical (or different) of could also be very informative for furthering the understanding of general cellular cholesterol homeostasis.

All together, we can suggest that in order to have a full and efficient action of the HDL or HDL-mimetics on the cholesterol efflux in macrophages we need to minimize the ABCA1 down-regulation and thus to find the right dose to avoid this down-regulation of ABCA1, the “gate-keeper” of cholesterol efflux from the cell. This balance would allow a cholesterol efflux toward the HDL acceptors and consequently would allow a decrease of the atherosclerotic plaque burden. Further clinical trials using HDL mimetics as well as with CETP inhibitors, which increase the HDL-C plasma concentration, would need to take into account this HDL-mediated down-regulation of ABCA1.

## Supporting Information

S1 Fig ABCA1 mRNA stimulated by cAMP decreases in J774 macrophages following CER-001 and HDL3 incubation. J774 macrophages were incubated for 24h with cAMP (300 μM) to increase the ABCA1 expression. cAMP was removed and CER-001, HDL3 and apoA-I at 250 μg/ml were added in the cell culture medium for 24h and ABCA1 mRNA level was determined by qPCR. The qPCR data represent the means of triplicate determinations from a single representative experiment out of 5 independent experiments.
(TIFF)
