# Supplementary figures and images for: HDL and CER-001 Inverse-Dose Dependent Inhibition of Atherosclerotic Plaque Formation in apoE-/- Mice: Evidence of ABCA1 Down-Regulation
Source: PLoS One. 2015 Sep 3;10(9):e0137584. doi: 10.1371/journal.pone.0137584 (PMC4559410; doi:10.1371/journal.pone.0137584)

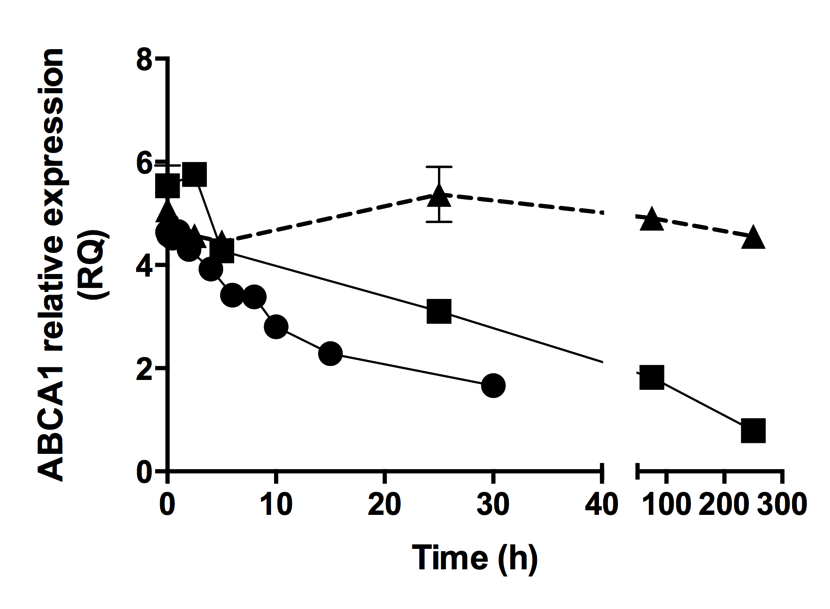

Supplement: S1 Fig — J774 macrophages were incubated for 24h with cAMP (300 μM) to increase the ABCA1 expression. cAMP was removed and CER-001, HDL3 and apoA-I at 250 μg/ml were added in the cell culture medium for 24h and ABCA1 mRNA level was determined by qPCR. The qPCR data represent the means of triplicate determinations from a single representative experiment out of 5 independent experiments. (TIFF) [file pone.0137584.s001.tiff]
